# Sequential activation of Elk-1/Egr-1/GADD45α by arsenic

**DOI:** 10.18632/oncotarget.1995

**Published:** 2014-05-22

**Authors:** Qiwen Shi, Vijaykumar Sutariya, Anupam Bishayee, Deepak Bhatia

**Affiliations:** ^1^ Department of Pharmaceutical Sciences, Northeast Ohio Medical University (NEOMED), Rootstown, Ohio, USA; ^2^ School of Biomedical Sciences, Kent State University, Kent, Ohio, USA; ^3^ Department of Pharmaceutical Sciences, University of South Florida, Tampa, Florida, USA; ^4^ Department of Pharmaceutical Sciences, American University of Health Sciences, Signal Hill, California, USA

**Keywords:** Egr-1, GADD45α, Arsenic, MAP kinase, Elk-1

## Abstract

Long-term exposure to arsenic, an environmental contaminant, leads to increased risks of cancers. In the present study, we investigated the sequential regulation of Elk-1 and Egr-1 on As^3+^-induced GADD45α, an effector of G2/M checkpoint. We found that As3+ transcriptionally induced both Elk-1 and Egr-1, and NF-κB binding site was necessary for As^3+^-induced Egr-1 promoter activity. However, specific inhibition of JNK, ERK, and Elk-1 inhibited Egr-1 induction. Furthermore, silencing of Egr-1 downregulated As^3+^-induced expression of GADD45α and ChIP assay confirmed the direct binding of Egr-1 to GADD45α promoter. Taken together, our data indicated that the increase of GADD45α in response to As^3+^ was mediated sequentially by Elk-1 and Egr-1.

## INTRODUCTION

Inorganic arsenic has a paradoxical role in cancer. Low-dose of arsenic is a treatment of some cancers like acute promyelocytic leukemia [1], while high-level of arsenic is a potential carcinogen, especially under chronic exposure. The molecular mechanisms of neither therapeutic effects nor carcinogenesis are fully understood. No evidence has shown that arsenic causes point mutations, however, the primary hypotheses explaining carcinogenic effects of arsenic include impairment of DNA repair, oncogene amplification, hypomethylation of DNA [2]. Furthermore, arsenic has been linked to cell growth and malignant transformation through sustained oxidative stress and aberrant kinase activation, including JNK, p38, checkpoint kinases, and Akt [3]. Arsenic has two biological important oxidation states: arsenate (As^5+^) and arsenite (As^3+^). As^3+^ is considerably more toxic than As^5+^, since it tends to react as a soft metal with thiols [4]. Human exposures to arsenic are mainly due to industrial activities such as the smelting process and coal burning, pollution in drinking water, contaminated food or drugs, and arsenic-containing dust [5].

Growth arrest and DNA damage inducible gene 45α (GADD45α) is a regulator at G_2_/M checkpoint, and plays roles in apoptosis, DNA damage response, and cell cycle arrest. It is often upregulated in response to various environmental stresses and drug therapies. The repression or deletion of GADD45α results in uncontrolled proliferation that is a survival mechanism [6]. The impairment of GADD45α (GADD45α-null mice) exhibits severe genomic instabilities, such as aneuploidy, chromosomal aberrations, gene amplification, centrosome amplification, abnormal mitosis and cytokinesis [7]. GADD45α interacts with proliferating cell nuclear antigen (PCNA) via competing with p21, which is a cyclin-dependent kinase inhibitor [8], MEKK4 that is an upstream of p38 and JNK pathways [9], histone core proteins [10], and FOXO3a [11]. Post-transcriptional regulation, including mRNA stabilization linked with nucleolin [12] and an internal ribosome entry site (IRES) [13], is another mechanism for the increase of GADD45α expression.

Both Early growth response 1 (Egr-1) and E-twenty-six (ETS)-like transcription factor 1 (Elk-1) are transcription factors. Egr-1 (also known as NGFI-A, zif 268, TIS8 and Krox-24) is rapidly and transiently induced by growth factors, differentiation signals [14] and ionizing radiation [15]. As a transcription factor that triggers transcription of multiple genes mediating cell growth and angiogenesis, the downstream of Egr-1 has been extensively studied. In lung cancer cell line A549, the upregulation of thrombospondin-1 (TSP-1), an anti-angiogenic and anti-invasion protein, upon cyclooxygenase (COX) inhibitors treatment is mediated by Egr-1 [16], and Egr-1 directly binds and downregulates stathmin expression which regulates the dynamics of microtubules [17]. During UV radiation, Egr-1 activates phosphatase and tensin (PTEN) homologue tumor suppressor [18]. Most of known Egr-1 targets are genes involved in tumor metastasis, such as plasminogen activator inhibitor-1 (PAI-1) [19], Transforming Growth Factor-beta 1 (TGF-β1) [20], and matrix metalloporteinase-9 (MMP-9) [21]. Elk-1 belongs to ETS-domain family and is involved in the regulation of cell growth, differentiation and survival. Its activation requires phosphorylation from upstreams.

In this study, we determined the roles of Elk-1 and Egr-1 in As^3+^-induced GADD45α expression. Our findings demonstrated that As^3+^ transcriptionally increased Egr-1 expression via ERK and JNK pathway, not Akt or p38 pathway, and the Nuclear Factor-kappa B (NF-κB) binding site was necessary to promote As^3+^-mediated Egr-1 transcriptional induction. However, As^3+^-induced Egr-1 expression and promoter activity were inhibited with Elk-1 downregulation. Furthermore, Egr-1 regulated As^3+^-induced GADD45α by direct binding to its promoter.

## RESULTS

### Induction of Egr-1 mRNA and Protein Expression by As^3+^ in BEAS-2B Cells

**Fig 1:**
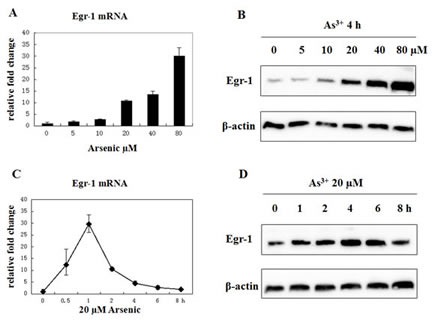
Arsenic treatment induces Egr-1 expression (A and B) BEAS-2B cells were treated with various concentrations of arsenic for 4 h, and mRNA expression was measured by real-time RT-PCR. The fold inductions correspond to the ratio between controls untreated and exposed cells (mean +/- SE). Egr-1 and β-actin protein expression were determined by western blot. (C and D) Cells were treated with 20 μM of As^3+^ for the time indicated, and mRNA level and protein expression were measured by the same methods above.

Human bronchial epithelial airway cell line, BEAS-2B, was selected to investigate the effect of As^3+^ because lung is the major target of arsenic-induced carcinogenesis, especially in occupational exposure. As^3+^ has already been proven to induce GADD45α in a dose-dependent manner [23]. Thus, we first needed to determine if As^3+^ would increase the expression of Egr-1 in BEAS-2B cells. BEAS-2B cells were incubated with 0, 5, 10, 20, 40, 80 μM of As^3+^ for 4 h, and protein expression and mRNA level were measured by real-time RT-PCR and western blot, respectively (Fig. 1A and 1B). A dose-dependent induction of Egr-1 expression by As^3+^ was seen, and 20 μM was chosen for later studies since previous report showed that exposure of BEAS-2B cells in a growing condition to 20 μM of As^3+^ resulted in a time-dependent increase in GADD45α mRNA and protein [13]. To examine whether the induction of Egr-1 occurs in a time-dependent manner, after treatment with 20 μM of As^3+^ at various time points, the expression of Egr-1 protein expression and mRNA level were measured. The induction of Egr-1 protein was time-dependent, and lagged behind the induction of Egr-1 mRNA of which the peak induction happened at 1 h after exposure (Fig. 1C). Egr-1 protein expression continued to increase at 1 to 4 h range, and was maintained until 8 h (Fig. 1D). These results confirm the increase of Egr-1 expression by As^3+^ in BEAS-2B cells.

### Functional analysis of As^3+^-mediated Egr-1 promoter induction

**Fig 2:**
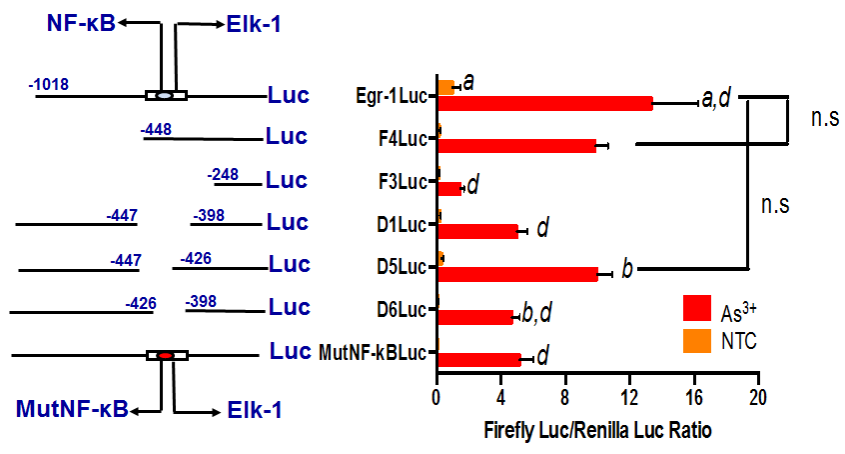
Arsenic transcriptionally regulates Egr-1 (Left) The scheme of full-length, deleted and mutated Egr-1 regulatory sequences. The numbers indicate the position of the deleted fragments relative to the transcription start site. (Right) BEAS-2B cells were transiently transfected with luciferase constructs, with or without arsenic treatment (right). The values are expressed as luciferase units, and data are represented as mean +/- SE. n=9, n.s: p > 0.1.

The induction of Egr-1 mRNA by As^3+^ suggested a relationship between As^3+^ -mediated Egr-1 induction and an increase in gene transcription. To confirm that Egr-1 is transcriptionally activated and to analyze the functional binding sites, we isolated and cloned 1.4 kb of genomic DNA located within Egr-1 5' regulatory sequences (pEgr-1-luc) which corresponds to the core promoter, plus the 5' noncoding region of the Egr-1 mRNA into a luciferase reporter gene and constructed multiple plasmids based on pEgr-1-luc. F4 and F3 promoter fragments were created by deleting 334 and 570 bp from the 5' end of pEgr-1-luc, respectively. The deletions of Δ1, Δ5, and Δ6 were precisely located at position -447/-397, -447/-426, -426/-397, respectively. A canonical NF-κB binding motif in the region deleted in Δ6 was revealed by computational sequence analysis, and thus we mutated this binding site (MutNF-κB). All these promoters were transfected into BEAS-2B cells that were then treated with or without 20 μM of As^3+^ overnight. The results showed a significant increase in the transcription activity of pEgr-1-luc, F4 and Δ5 constructs, while F3 remained unresponsive, and Δ6 and MutNF-κB partially abolished the Egr-1 promoter response to As^3+^ (Fig. 2). These results demonstrate that NF-κB binding site located at -425/-417 is necessary but not sufficient to promote As^3+^-mediated Egr-1 transcriptional induction.

### The regulation of As^3+^-induced Egr-1 expression by MAPK and Akt

**Fig 3:**
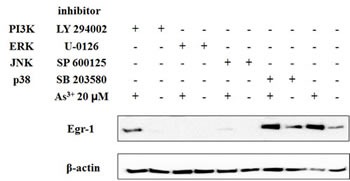
ERK and JNK pathway is required for induction of Egr-1 by As^3+^ BEAS-2B cells were pretreated with 10 μM of SB 203580, LY 294002, U 0126, and 20 μM of SP 600125 for 1 h and then with or without arsenic for 4 h. Cells were isolated for protein followed by western blot analysis for Egr-1 and β-actin.

The mitogen-activated protein kinase (MAPK) cascade is considered to be a major signaling pathway that links signals from the cell surface to the nuclear events triggered by various stimuli [24], and there are three types of mammalian MAP kinases: JNK, ERK, and p38 [25]. The PI3K/Akt pathway modulates the function of a number of substrates involved in the regulation of cell survival, cell cycle progression and cellular growth [26]. To identify the involvement of MAPK and Akt pathways in As^3+^-mediated Egr-1 induction, BEAS-2B cells were pretreated with 10 μM of p38 inhibitor (SB 203580), 20 μM of JNK inhibitor (SP 600125), 10 μM of ERK inhibitor (U-0126), and 10 μM of PI3K inhibitor (LY 294002) for 1 h, respectively, followed by with or without 20 μM of As^3+^ treatment for 4 h. The Egr-1 expression was measured by western blot. The data demonstrates that SP 600125 and U-0126 blocked As^3+^-mediated Egr-1 induction in BEAS-2B cells whereas SB 203580 and LY 294002 (Fig. 3) had no effect.

### Activation of Elk-1 by As^3+^

**Fig 4:**
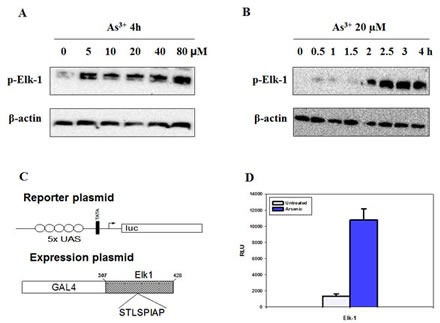
Elk-1 is activated by As^3+^ (A and B) Cells were treated with various concentrations of As^3+^ for different time points. Protein expression was analyzed by western blot. (C) A modular structure of GAL4 Elk-1 shows the reporter plasmid pUAS5 luc containing luciferase reporter gene, a TATA box and five binding sites for GAL4 (UAS) upstream of the TATA box. (D) BEAS2B cells were co-transfected with the reporter plasmid and the expression vector, and maintained in complete medium for 48 h followed by As^3+^ treatment for additional 12 h. The luciferase activity of the cell lysates was measured. Data are represented as mean +/- SE.

Elk-1 is in a transcriptionally active state when phosphorylated by the MAPK cascade [27]. In this report, we found that As^3+^ augmented Elk-1 phosphorylation in a dose- and time-dependent way (Fig. 4A and 4B). Elk-1 phosphorylation reached maximal at 2 h, which was earlier than the peak induction of Egr-1 protein. The promoter activity of Elk-1 was also investigated. A GAL4-Elk-1 expression plasmid, which contains the NH_2_ terminal 147 amino acids of the yeast transcription factor GAL4 encompassing the DNA binding domain and the activation domain of Elk-1, was co-transfected into BEAS-2B cells together with a report plasmid constructed with GAL4-binding sequences (Fig. 4C). As shown in Fig. 4D, As^3+^ increased the trans-acting activity of GAL4-Elk-1 by about 10 folds. These data propose the activation of Elk-1 by As^3+^.

### The requirement of Elk-1 activation for As^3+^-induced Egr-1 synthesis

**Fig 5:**
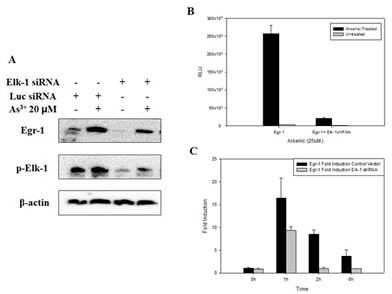
Inhibition of Elk-1 by shRNA or siRNA inhibits Egr-1 protein expression, promoter activity and mRNA synthesis (A) Cells were transfected with Elk-1 siRNA for 48 h followed by As^3+^ treatment overnight. (B) Cells were transfected with Elk-1 shRNA for 24 h, and then transfected with Egr-1 promoter for 48 h with following treatment of As^3+^ overnight. (C) As^3+^ was added 48 h after Elk-1 shRNA transfection, and mRNA was extracted as time indicated. Real-time RT-PCR was performed to detect mRNA level. Data are represented as mean +/- SE.

As shown previously, there is a Elk-1 binding site in Egr-1 promoter [28], and in C6 glioma cells, ERβ-activated Egr-1 transcription is through ERK/Elk-1 pathway [29]. Thus, to confirm the relationship between Elk-1 and Egr-1, we transfected BEAS-2B cells with Elk-1 siRNA or shRNA followed by As^3+^ treatment. The induction of Egr-1 protein expression by As^3+^ was diminished along with the knockdown of Elk-1 (Fig. 5A), as well as the induction of Egr-1 promoter activity which only expressed 10% luciferase compared to As^3+^ only control (Fig. 5B). The increase of Egr-1 mRNA by As^3+^ was also blocked by Elk-1 shRNA. The basal Egr-1 mRNA level was not affected by Elk-1 shRNA, however, 1 h induction of Egr-1 mRNA by As^3+^ was reduced by 50% when transfected with Elk-1 shRNA, and after 1 h, Egr-1 mRNA dropped back to basal level in Elk-1 shRNA transfected cells (Fig. 5C). These results indicate that As^3+^-induced Egr-1 synthesis is mediated by the increased activation of Elk-1.

### The contribution of Egr-1 to GADD45α expression upon As^3+^ treatment

**Fig 6:**
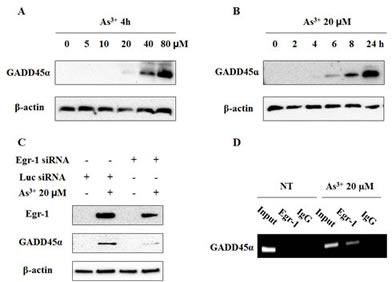
Egr-1 transcriptionally regulates As-induced GADD45α expression (A and B) Cells were treated with As^3+^ as indicated doses and hours. (C) Egr-1 siRNA blocks the induction of GADD45α by As^3+^. BEAS-2B cells were transiently transfected for 48 h in Opti-MEM with Egr-1 siRNA or Luciferase siRNA as indicated, and then treated overnight with or without 20 μM of As^3+^. Cells were isolated for protein followed by western blot analysis for Egr-1, GADD45α, and β-actin. (D) ChIP experiment on As^3+^-treated or non-treated BEAS-2B cells. Chromatin extracts were immunoprecipitated with specific antibodies to Egr-1 or normal rabbit IgG (control). The detection of GADD45α promoters was performed by PCR.

Egr-1 regulates GADD45α promoter activities by direct binding to its endogenous regulatory sequence upon UV irradiation in an immortal human keratinocyte cell line (HaCaT) [22]. Given that the increase of GADD45α protein expression occurred after 6 h exposure to As^3+^ (Fig. 6A and B), which is later than that of Egr-1 under similar condition, we considered GADD45α as a target of Egr-1 in BEAS-2B cells when treated with As^3+^. To test whether Egr-1 is involved in the regulation of As^3+^-induced GADD45α expression, we transiently transfected cells with Egr-1 siRNA before As^3+^ treatment. The suppression of Egr-1 by specific siRNA of Egr-1 blocked the induction of GADD45α protein expression by As^3+^ (Fig. 6C). Therefore, Egr-1 is the upstream of As^3+^-induced GADD45α. To further investigate whether Egr--1 protein directly binds to GADD45α promoter, we performed a Chromatin immunoprecipitation (ChIP) experiment on BEAS-2B cells treated with or without 20 μM of As^3+^. The detection of the GADD45α promoter was performed by PCR analysis with specific primer pairs located in the core promoter and the 5' noncoding region. Efficiency of the primer was tested on the genomic DNA input. The results depicted significant amplification of GADD45α promoter in chromatin under As^3+^ treatment when immunoprecipitated with anti-Egr-1. In the control group (IgG) (Fig. 6D) no amplification was observed. Taken together, our data indicate direct binding of Egr-1 protein to GADD45α promoter in BEAS-2B cells when treated with As^3+^, thus confirming the role of Egr-1 in the regulation of As^3+^-induced GADD45α expression.

## DISCUSSION

As^3+^ likely acts as a tumor promoter rather than a direct mutagen [30]. Similar to heat shock, sodium arsenite activates Egr-1 via the JNK1 and p38 signal transduction pathways in mouse embryo fibroblast cell line NIH3T3 [31], indicating As^3+^-induced stress at the molecular level shares many features with the heat shock response, and produces oxidative stress at least. In contrast, our data shows that JNK and ERK, instead of p38, activate Egr-1 when treated with As^3+^. The retinoid 6-[3-(1-adamantyl)-4-hydroxyphenyl]-2-naphthalene carboxylic acid (AHPN), a cell proliferation inhibitor and apoptosis inducer has been shown to induce Egr-1 expression through ERK1/2 signaling pathway rather than activation of p38 in lung cancer cells [32]. Egr-1 exhibits either oncogenic or tumor suppressive properties depending on the type of cells and stimuli. Blocking nuclear translocation of Egr-1 is responsible for the loss of PTEN expression in gefitinib-resistant lung cancer cells [33]. Egr-1 also mediates TNF-α induction in bystander response in A549 cells, and plays an essential role in eliciting bystander-mediated apoptotic response in the same cell line [34]. Conversely, Egr-1, which is induced by ERK pathway activation, induces and enhances vascular endothelial growth factor-A (VEGF-A) in lung cancer [35]. Here, in our research, Egr-1 performs as a tumor suppressor and leads to cell growth arrest and apoptosis via ERK and JNK in arsenic treated BEAS-2B cells.

**Fig 7:**
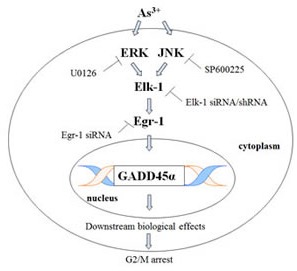
As^3+^ sequentially activates MAPK cascades, Elk-1, Egr-1.and GADD45α This scheme is a summary of Elk-1/Egr-1/GADD45α pathway. Egr-1 directly binds GADD45α promoter and increases the expression of GADD45α, which results in subsequent biological events and finally arrests cell cycle progression.

Mutation of NF-κB binding site in Egr-1 promoter totally eliminates UVB-mediated Egr-1 transcriptional induction in epidermal cells, as well as Δ6 promoter [22]. However, our luciferase assay exhibits a partial inhibition in As^3+^-mediated Egr-1 transcriptional induction. An Elk-1 binding site was found by computational sequence analysis in the deleted region near NF-κB binding site, and the importance of Elk-1 binding site has been pointed by many previous studies. Moreover, the involvement of ERK in Egr-1 induction also implies the role of Elk-1 binding site in Egr-1 transcriptional activation. Lipopolysaccharide-induced Egr-1 activation is regulated by Elk-1 phosphorylation through the MEK/ERK signaling cascade [36], and it requires the recruitment of SRF to SREs in the Egr-1 promoter (43 IGFBP). In prostate tumors, Egr-1 expression is frequently upregulated, and Egr-1 expression in prostate cancer cells PC3 is mediated through an EGF-ERK-Elk-1 signaling cascade [37]. Hemin activates Elk-1 and NF-κB and promotes their interaction with Egr-1 promoter [38]. In this study, we confirmed the indispensability of Elk-1 activation in As^3+^-induced Egr-1 activity by showing that Egr-1 activity is abolished when Elk-1 is downregulated.

GADD45α expression is normally low and is activated in response to various stresses as a protective mechanism in normal cells. High level of GADD45α arrests cell cycle progression at G2/M checkpoint and repairs DNA by nucleotide excision [39]. An 8% increase in G2/M phase was observed when treated with 10 μM of As^3+^ for 24 h in BEAS-2B cells (Data not shown), suggesting the function of GADD45α in stress-insulted normal cells. In addition, GADD45α expression is aberrant in cancers [40-41], proposing that GADD45α could be a potential therapeutic target. Therefore, knowing how GADD45α is regulated is essential to design GADD45α-targeted treatment. Currently, we are also working at GADD45α-targeted gene therapy, which showed its effect in A549 cells (Unpublished data, Shi, Q). Besides Egr-1, we also found that the rise of activating factor 3 (ATF3) expression by As^3+^ was later than that of Egr-1, but earlier than that of GADD45α (data not shown), implying a possible sequential activation of these three proteins. Previous study showed that the expression of ATF3 occurred via Egr-1 downstream of ERK1/2 [42]. It is likely that As^3+^ could induce GADD45α via Egr-1/ATF3 pathway.

In conclusion, our study is the first time demonstration of MAPK/Elk-1/Egr-1/GADD45α pathway in normal bronchial cells. This sequential activation of MAPK/Elk-1/Egr-1/GADD45α gives an explanation to the carcinogenic effect of As^3+^ and also elucidates the regulation of GADD45α during carcinogenesis.

## MATERIALS AND METHODS

### Cell culture and cell treatment

The human bronchial epithelial cell line (BEAS-2B) was purchased from ATCC (Manassas, VA) and maintained in Dulbecco's modified Eagle's medium (DMEM) supplemented with 10% fetal bovine serum and grown at 37 °C, 5% CO_2_ in a humidified incubator. The BEAS-2B cells were seeded in 6-well tissue plates at a density of 1 × 10^6^ cells/well and cultured for 24 h. The cells were treated with the indicated concentrations of arsenic chloride (As^3+^) (Johnson Matthey, MI) for indicated time with or without 1 h pretreatment of p38 inhibitor (SB 203580), JNK inhibitor (SP 600125), ERK inhibitor (U-0126), and Akt inhibitor (LY 294002) (Santa Cruz Biotechnology, Inc., TX).

### Real-time RT-PCR

Total RNA was prepared using Column-Pure^TM^ total tissue RNA isolation kit (LAMDA Biotech, Inc. St. Louis, MO) according to the manufacture's recommendation, RNA was quantitated by UV spectroscopy and stored in RNase-free H_2_O at -80 °C. Reserve transcription reactions were carried by using AccuPower^®^ RT PreMix (Bioneers, Santa Fe, NM) following the manufacture's protocols. Real-time PCR was carried out using Applied Biosystems^®^ 7900HT fast Real-time PCR system (Life technologies, Grand Island, NY). The cDNA generated from reverse transcription was diluted 1:10 and 5μl was used to conduct PCR. PCR reactions were carried out in microAmp^®^ fast optical 96-well reaction plates (Life technologies, Grand Island, NY; Maxima SYBR Green/ROX qPCR master mix (2X) (Thermo Scientific), forward and reverse primers (0.3 μM) (Eurofins) in a final PCR reaction volume of 20 μl. Amplification parameters were: denaturation at 95 °C 10 min, followed by 40 cycles of 95 °C, 15 s; 60 °C, 60 s. Samples were analyzed in duplicate, and GAPDH was used as an endogenous control. Fold induction was calculated using the formula 2-ΔΔCt, where ΔCt = target gene Ct - GAPDH Ct, and ΔΔCt is based on the mean ΔCt of respective control (non-arsenic treated).

### Western blot analysis

The cells were washed with PBS, and then lysed using Lysis-M reagent (Roche Diagnostics, IN) containing 1 × phosphatase inhibitors cocktail I and 1 × protease inhibitor cocktail II (Boston BioProducts, MA). Plates were scraped and samples were sonicated for 10 rounds at 30% duty cycle, 3-output control. Quantitation of proteins was done using the bicinchoninic acid (BCA) protein-assay kit from Pierce (Rockford, IL). Protein extracts were separated by electrophoresis on a 4-20% Express PAGE gel (GenScript, NJ) and transferred to 0.45-μm-pore size nitrocellulose membrane (Thermo scientific, IL). The filters were preincubated for 40 min in 1 × Tris-Buffered Saline and Tween 20 (TBST) (Santa Cruz Biotechnology, Inc., TX) and 4% dry milk, and then cut based on protein markers, and subsequently sealed for overnight incubation at 4 °C respectively with the antibody Egr-1 (588), GADD45α (H-165), p-Elk-1 (B-4) and β-actin (C4) (Santa Cruz Biotechnology, Inc., TX), diluted 1:10,000 or 1:200 according to manufacture's recommendation in 1 × TBST and 5% Bovine Serum Albumin (BSA) plus 0.02% NaN_3_. After washing 3 × 5 min in 1 × TBST, the membranes were incubated with a secondary donkey anti-rabbit IgG or goat anti-mouse lgG conjugated to peroxidase and diluted 1:5,000 in 1 × TBST 4% dry milk. Detection was carried out using the West Pico from SuperSignal (Thermo Scientific, IL).

### Dual luciferase assay

Plasmids were constructed as described by Thyss et al [22]. The BEAS-2B cells were seeded in 96-well plate at a density of 30,000 cells/well in Opti-MEM® containing 10% fetal bovine serum and 1% NEAA without antibiotics for 24 h. Then 0.2 μg of target plasmid and 0.02 μg of Renilla luciferase control reporter vector (pRL) were co-transfected with the complexes consist of 0.3 μl of Lipofectamine^TM^ 2000 (Life technologies, Grand Island, NY) and 0.3 μl of CombiMag reagent (Boca Scientific, Boca Raton, FL), followed by incubation on the magnetic plate at 37 °C in a CO_2_ incubator for 20 min. After 48 h, cells were treated with 20 μM of As^3+^ overnight, and then lysed by M-PER^®^ Mammalian Protein Extraction Reagent (Thermo Scientific, IL). Luciferase activity was measured using Dual-luciferase Reporter Assay System (Promega).

### siRNA/shRNA transfection

BEAS-2B cells were seeded in 6-well plate at a density of 1 × 10^6^ cells/well in Opti-MEM^®^ containing 10% fetal bovine serum and 1% NEAA without antibiotics for 24 h, and then transfected with Egr-1 siRNA, Elk-1 siRNA/shRNA or luciferase siRNA (Bioneers, Alameda, CA) using complexes formed by 5 μl of Lipofectamine^TM^ 2000 (Life technologies, Grand Island, NY) and 2.5 μl of CombiMag reagent (Boca Scientific, Boca Raton, FL) followed by incubating on the magnetic plate at 37 °C in a CO_2_ incubator for 20 min. After 48 h, cells were treated with 20 μM of As^3+^ overnight, and total proteins were extracted for western blot or real-time RT-PCR. Or after 24 h, cells were transfected with Egr-1 promoter followed by same treatment and the lysates were detected by luciferase assay.

### Chromatin immunoprecipitation assay

Chromatin immunoprecipitation (ChIP) assay was performed using ChIP Assay Kit (Upstate Biotechnology, Charlottesville, VA). In concise, 1 × 10^7^ cells were cross-linked with 1% formaldehyde quenched by 1 × Glycine at room temperature for 10 min. Then cells were washed with ice-cold PBS, harvested by scraping and resuspended in lysis buffer containing protease inhibitor cocktail II. After incubation for 15 min on ice with vortex every 5 min, cells were sonicated to generate about 500bp DNA fragments, and centrifuged for 10 min at 4 °C. The supernatants were incubated with protein A magnetic beads and anti-Egr1 (Santa Cruz) or IgG at 4 °C overnight with rotation. The immune complexes then were washed and eluted with the ChIP elution buffer and reverse cross-linked by heating at 62 °C for 2 h followed by 95 °C for 10 min. Before immunoprecipitate, a small chromatin-protein sample was excluded and used as input sample for a positive control for the PCR reaction. DNA was extracted by Chromatin IP DNA Purification kit (Active Motif) according to manufacture's instruction. PCR was performed using the following primers: forward primer: 5'-GGCGGAAGGTGGTTGGCTGA-3', reverse primer: 5'-AGCTCAGGCCCTGGCGCTCT-3', at 62 °C for 33 cycles.

## References

[1] Lengfelder E, Hofmann WK, Nowak D (2012). Impact of arsenic trioxide in the treatment of acute promyelocytic leukemia. Leukemia.

[2] Rudel R, Slayton TM, Beck BD (1996). Implications of arsenic genotoxicity for dose response of carcinogenic effects. Regul Toxicol Pharmacol.

[3] Liu J, Chen B, Lu Y, Guan Y, Chen F (2012). JNK-dependent Stat3 phosphorylation contributes to Akt activation in response to arsenic exposure. Toxicol Sci.

[4] Yang HC, Fu HL, Lin YF, Rosen BP (2012). Pathways of arsenic uptake and efflux. Curr Top Membr.

[5] Roy P, Saha A (2002). Metabolism and toxicity of arsenic: A human carcinogen. Curr Sci.

[6] Rosemary Siafakas A, Richardson DR (2009). Growth arrest and DNA damage-45 alpha (GADD45alpha). Int J Biochem Cell Biol.

[7] Hollander MC, Sheikh MS, Bulavin DV, Lundgren K, Augeri-Henmueller L, Shehee R, Molinaro TA, Kim KE, Tolosa E, Ashwell JD, Rosenberg MP, Zhan Q, Fernandez-Salguero PM, Morgan WF, Deng CX, Fornace AJ (1999). Genomic instability in Gadd45a-deficient mice. Nat Genet.

[8] Smith ML, Chen IT, Zhan Q, Bae I, Chen CY, Gilmer TM, Kastan MB, O'Connor PM, Fornace AJ (1994). Interaction of the p53-regulated protein Gadd45 with proliferating cell nuclear antigen. Science.

[9] Takekawa M, Saito H (2001). Involvement of the Oct-1 regulatory element of the gadd45 promoter in the p53-independent response to ultraviolet irradiation. Cancer Res.

[10] Carrier F, Georgel PT, Pourquier P, Blake M, Kontny HU, Antinore MJ, Gariboldi M, Myers TG, Weinstein JN, Pommier Y, Fornace AJ (1999). Gadd45, a p53-responsive stress protein, modifies DNA accessibility on damaged chromatin. Mol Cell Biol.

[11] Brunet A, Sweeney LB, Sturgill JF, Chua KF, Greer PL, Lin Y, Tran H, Roos SE, Mostoslavsky R, Cohen HY, Hu LS, Cheng HL, Jedrychowski MP, Gyqi SP, Sinclair DA, Alt FW (2004). Stress-dependent regulation of FOXO transcription factors by the SIRT1 deacetylase. Science.

[12] Zhang Y, Bhatia D, Xia H, Castranova V, Shi X, Chen F (2006). Nucleolin links to arsenic-induced stabilization of GADD45alpha mRNA. Nucleic Acids Res.

[13] Chang Q, Bhatia D, Zhang Y, Meighan T, Castranova V, Shi X, Chen F (2007). Incorporation of an internal ribosome entry site-dependent mechanism in arsenic-induced GADD45 alpha expression. Cancer Res.

[14] Sukhatme VP, Cao XM, Chang LC, Tsai-Morris CH, Stamenkovich D, Ferreira PC, Cohen DR, Edwards SA, Shows TB, Curran T, Le Baeu MM, Adamson ED (1998). A zinc finger-encoding gene coregulated with c-fos during growth and differentiation, and after cellular depolarization. Cell.

[15] Datta R, Rubin E, Sukhatme V, Qureshi S, Hallahan D, Weichselbaum RR, Kufe DW (1992). Ionizing radiation activates transcription of the EGR1 gene via CArG elements. Proc Natl Acad Sci U S A.

[16] Moon Y, Bottone FG (2005). McEntee MF, Eling TE. Suppression of tumor cell invasion by cyclooxygenase inhibitors is mediated by thrombospondin-1 via the early growth response gene Egr-1. Mol Cancer Ther.

[17] Fang L, Min L, Lin Y, Ping G, Rui W, Ying Z, Xi W, Ting H, Li L, Ke D, Jihong R, Huizhong Z (2010). Downregulation of stathmin expression is mediated directly by Egr-1 and associated with p53 activity in lung cancer line A549. Cell Signal.

[18] Virolle T, Krones-Herzig A, Baron V, Birle D, Mercola D, Mustelin T, de Belle I (2001). The Egr-1 transcription factor directly activates PTEN during irradiation-induced signalling. Nat Cell Biol.

[19] Shin SY, Ko J, Chang JS, Min DS, Choi C, Bae SS, Kim MJ, Hyun DS, Kim JH, Han MY, Kim YH, Kim YS, Na DS, Suh PG, Lee YH (2002). Negative regulatory role of overexpression of PLC gamma 1 in the expression of early growth response 1 gene in rat 3Y1 fibroblasts. FASEB J.

[20] Shingu T, Bornstein P (1994). Overlapping Egr-1 and Sp1 sites function in the regulation of transcription of the mouse thrombospondin 1 gene. J Biol Chem.

[21] Shin SY, Kim JH, Baker A, Lim Y, Lee YH (2010). Transcription factor Egr-1 is essential for maximal matrix metalloproteinase-9 transcription by tumor necrosis factor alpha. Mol Cancer Res.

[22] Thyss R, Virolle V, Imbert V, Peyron JF, Aberdam D, Virolle T (2005). NF-kappaB/Egr-1/Gadd45 are sequentially activated upon UVB irradiation to mediate epidermal cell death. EMBO J.

[23] Bower JJ, Leonard SS, Chen F, Shi X (2006). As(III) transcriptionally activates the gadd45a gene via the formation of H202. Free Radic Biol Med.

[24] Seger R, Krebs EG (1995). The MAPK signaling cascade. FASEB J.

[25] Johnson GL, Lapadat R (2002). Mitogen-activated protein kinase pathways mediated by ERK, JNK and p38 protein kinases. Science.

[26] Fresno Vara JA, Casado E, de Castro J, Cejas P, Belda-Iniesta C, Gonzalez-Baron M (2004). PI3K/Akt signaling pathway cancer. Cancer Treat Rev.

[27] Mut M, Lule S, Demir O, Kurnaz IA, Vural I (2012). Both mitogen-activated protein kinase (MAPK)/extracellular-signal-regulated kinases (ERK) 1/2 and phosphatidylinositide-3-OH kinase (PI3K)/Akt pathways regulate activation of E-twenty-six (ETS)-like transcription factor 1 (Elk-1) in U138 glioblastoma cells. Int J Biochem Cell Biol.

[28] Watson DK, Robinson L, Hodge DR, Kola I, Papas TS, Seth A (1997). FLI1 and EWS-FLI1 function as ternary complex factors and ELK1 and SAP1a function as ternary and quaternary complex factors on the Egr1 promoter serum response elements. Oncogene.

[29] Kim J, Jeong I, Lim Y, Lee Y, Shin SY (2011). Estrogen receptor β stimulates Egr-1 transcription via MEK1/Erk/Elk-1 cascade in C6 glioma cells. JBMB.

[30] Bernstam L, Nriagu J (2000). Molecular aspects of arsenic stress. J Toxico Environ Health B Crit Rev.

[31] Lim CP, Jain N, Cao X (1998). Stress-induced immediate-early gene, egr-1, involves activation of p38/JNK1. Oncogene.

[32] Sakaue M, Adachi H, Dawson M, Jetten AM (2001). Induction of Egr-1 expression by the retinoid AHPN in human lung carcinoma cells is dependent on activated ERK1/2. Cell Death Differ.

[33] Yamamoto C, Basaki Y, Kawahara A, Nakashima K, Kage M, Izumi H, Kohno K, Uramoto H, Yasumoto K, Kuwano M, Ono M (2010). Loss of PTEN expression by blocking nuclear translocation of EGR1 in gefitinib-resistant lung cancer cells harboring epidermal growth factor receptor-activating mutations. Cancer Res.

[34] Shareef MM, Cui N, Burikhanov R, Gupta S, Satishkumar S, Shajahan S, Mohiuddin M, Rangnekar VM, Ahmed MM (2007). Role of tumor necrosis factor-alpha and TRAIL in high-dose radiation-induced bystander signaling in lung adenocarcinoma. Cancer Res.

[35] Shimoyamada H, Yazawa T, Sato H, Okudela K, Ishii J, Sakaeda M, Kashiwaqi K, Suzuki T, Mitsui H, Woo T, Tajiri M, Ohmori T, Ogura T, Masuda M, Oshiro H, Kitamura H (2010). Early growth response-1 induces and enhances vascular endothelial growth factor-A expression in lung cancer cells. Am J Pathol.

[36] Guha M, O'Connell MA, Pawlinski R, Hollis A, McGovern P, Yan SF, Stern D, Mackman N (2001). Lipopolysaccharide activation of the MEK-ERK1/2 pathway in human monocytic cells mediates tissue factor and tumor necrosis factor alpha expression by inducing Elk-1 phosphorylation and Egr-1 expression. Blood.

[37] Gregg J, Fraizer G (2011). Transcriptional regulation of EGR1 by EGF and the ERK signaling pathway in prostate cancer cells. Genes Cancer.

[38] Hasan RN, Schafer AL (2008). Hemin upregulates Egr-1 expression in vascular smooth muscle cells via reactive oxygen species ERK-1/2-Elk-1 and NF-kappaB. Circ Res.

[39] Hollander CM, Fornace AJ (2002). Genomic instability, centrosome amplification, cell cycle checkpoints and Gadd45a. Oncogene.

[40] Higashi H, Vallbohmer D, Warnecke-Eberz U, Hokita S, Xi H, Brabender J, Metzqer R, Baldus SE, Natsuqoe S, Aikou T, Holscher AH, Schneider PM (2006). Down-regulation of Gadd45 expression is associated with tumor differentiation in non-small cell lung cancer. Anticancer Res.

[41] Al-Romaih K, Sadikovic B, Yoshimoto M, Wang Y, Zielenska M, Squire JA (2008). Decitabine-induced demethylation of 5' CpG island in GADD45A leads to apoptosis in osteosarcoma cells. Neoplasia.

[42] Brunet A, Sweeney LB, Sturgill JF, Chua KF, Greer PL, Lin Y, Tran H, Ross SE, Mostoslavsky R, Cohen HY, Hu LS, Cheng HL, Jedrychowski MP, Gyqi SP, Sinclair DA, Alt FW, Greenberg ME (2004). Stress-dependent regulation of FOXO transcription factors by the SIRT1 deacetylase. Science.

